# Rhythmic Leptin Is Required for Weight Gain from Circadian Desynchronized Feeding in the Mouse

**DOI:** 10.1371/journal.pone.0025079

**Published:** 2011-09-19

**Authors:** Deanna Marie Arble, Martha Hotz Vitaterna, Fred W. Turek

**Affiliations:** Northwestern University, Center for Sleep and Circadian Biology, Evanston, Illinois, United States of America; Vanderbilt University, United States of America

## Abstract

The neuroendocrine and metabolic effects of leptin have been extensively researched since the discovery, and the later identification, of the leptin gene mutated within the *ob/ob* mouse. Leptin is required for optimal health in a number of physiological systems (e.g. fertility, bone density, body weight regulation). Despite the extensive leptin literature and many observations of leptin’s cyclical pattern over the 24-hour day, few studies have specifically examined how the circadian rhythm of leptin may be essential to leptin signaling and health. Here we present data indicating that a rhythmic leptin profile (e.g. 1 peak every 24 hours) leads to excessive weight gain during desynchronized feeding whereas non-rhythmic leptin provided in a continuous manner does not lead to excessive body weight gain under similar feeding conditions. This study suggests that feeding time can interact with leptin’s endogenous rhythm to influence metabolic signals, specifically leading to excessive body weight gains during ‘wrongly’ timed feeding.

## Introduction

The master circadian clock, located within the surprachiasmtic nuclei (SCN), orchestrates the cyclic rhythms of many behaviors, tissues, hormones, genes, and other physiological processes. Studies over the last decade have demonstrated that the molecular clock mechanism operates in a cell autonomous fashion in most, if not all, peripheral tissues [1], [2], [3]. Under normal conditions, peripheral clocks remain synchronized to the 24-hour day by the SCN. However, during times of desynchronization, peripheral tissues can become out of phase with the external light:dark cycle, the SCN, and other tissues [3]. Such desynchronization, as occurs during classic restricted feeding and desynchronized feeding (DF), could lead to cardiometabolic health consequences [4].

In both humans and rodents, feeding during the ‘wrong’ circadian time has been implicated in body weight gain. Night-shift workers, who must be awake, active, and eating during the night are at a higher risk for obesity and cardiometabolic diseases [5], [6]. Nocturnal rats placed in a slowly rotating wheel for 8 hours during the day (e.g. a shift-work schedule) show a loss of glucose rhythmicity, an inverted triglyceride rhythm, and an increase in body weight compared to ‘day’ working rats [7]. Even without forced activity, mice eating a high-fat diet only during the ‘wrong’ circadian phase (e.g. the 12-hour light phase) gain 2.5x more weight than mice fed the same diet during the mouse’s natural feeding period [8]. Additional studies have further identified that a high-fat diet consumed during the later portion of the active period, as opposed to the early portion, results in body weight gain and signs of the metabolic syndrome [9]. It is hypothesized that eating during the ‘wrong’ circadian time contributes to circadian desynchronization and increased weight gain.

With nocturnal rodents, a desynchronized feeding (DF) protocol [8] uses two conflicting environmental stimuli to create circadian desynchronization: one from the light:dark cycle, which works in unison with the central circadian clock to cause rest and fasting during the light phase; and the other from behavioral feeding, which is experimentally restricted only to the circadian light phase. In this way, DF acts as a metabolic and circadian challenge by restricting access to a palatable high-fat diet to the ‘wrong’ circadian phase, going against cues from the light:dark cycle and the circadian clock to fast and rest.

A wild type B6 mouse challenged with DF will gain significantly more weight than a B6 mouse fed only during the normal circadian feeding phase (i.e. synchronized feeding, SF)[8]. The mechanisms that lead to excessive weight gain from DF are currently unknown. The present study tests the hypotheses that leptin plays an important role in weight gain during DF and additionally, that the circadian expression of leptin itself influences that weight gain.

Since its discovery, leptin has been indicated as an important component of metabolism, reproduction, motivated behaviors, and others [10]. Leptin is secreted by adipose tissue in proportion to body fat amount and relays fat storage information to the brain. When leptin is given peripherally, feeding is reduced, reflecting leptin’s role in feeding behavior and energy balance [11]. Obese individuals have accompanying high leptin levels and are generally considered leptin resistant due to the ineffectiveness of leptin treatment to decrease body weight [12].

Naturally occurring fluctuations in leptin levels occur over the period of a day and are closely associated with feeding and insulin release in both humans and rodents [13]. Notably, when meals are shifted by 6.5 hours in humans, leptin expression also shifts by 5–7 hours in the same direction [14]. Leptin’s tight coupling with feeding could suggest that leptin may be able to communicate meal timing information to the brain in addition to fat storage information. The leptin rhythm itself may also be influenced directly by the circadian clock. Normally, the circadian clock dictates the nocturnal nature of mouse feeding behavior, and feeding in turn influences leptin expression. However, under constant and continuous feeding conditions, a circadian leptin rhythm persists [15], suggesting an endogenous cycle independent of meal timing. Conversely, long periods of fasting have been noted to lead to a reduction in leptin levels and eliminate the leptin diurnal rhythm [13]. A circadian misalignment between behavior and circadian timing leads to lower overall leptin levels [16] suggesting that leptin responds to the endogenous circadian clock independently of some behaviors like feeding [17]. Additionally, evidence from cultured adipocycte cells suggests the presence of a circadian rhythm of leptin secretion influenced by the endogenous circadian clock of the adipocycte [18]. Leptin has also been found to advance the master circadian clock, the SCN, *in vitro* suggesting a bidirectional relationship between the circadian clock and leptin [19]. Together, these data suggest that the circadian leptin rhythm may provide a bridge between feeding cues, metabolic state, and circadian timing.

Despite a wealth of literature describing the natural circadian variation in leptin over the 24-hour day, surprisingly few studies have examined the functional significance of the circadian leptin rhythm for health and treatment (e.g. chronotherapy) [20]. To specifically examine the significance of a leptin rhythm, the present study uses the *ob/ob* mouse, on a C57BL/6J (B6) genetic background, which has a mutation in the leptin gene rendering it ineffective throughout the mouse. As a result of the absence of leptin, *ob/ob* mice are hyperphagic, obese, and develop characteristics associated with the metabolic syndrome in humans including elevated glucose, and insulin insensitivity [21]. By using this mouse model with no endogenous leptin, we are able to create a leptin rhythm with peaks occurring at any circadian time by using injections, or conversely, a complete absence of rhythm by using a constant release mini-osmotic pump. In the present study, we use the *ob/ob* mouse to test the hypothesis that non-rhythmic continuous leptin will have differential effects on body weight gain compared to rhythmic leptin. We present data indicating that the presence of leptin alone is not sufficient to lead to weight gain from DF, instead, the presence of leptin and a rhythmic peaking of leptin is necessary for weight gain to occur from DF suggesting that the endogenous leptin rhythm is a possible mechanism leading to excessive weight gains from ‘wrongly’ timed feeding.

## Results

**Figure 1:**
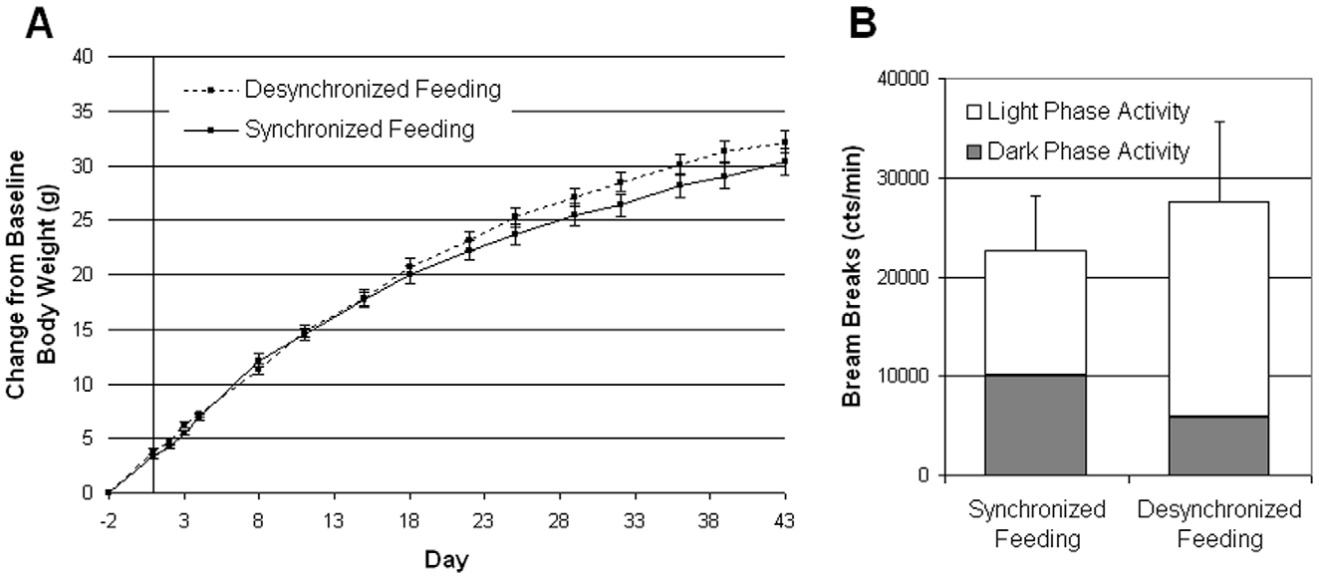
Experiment 1: Body weight gain and total activity in leptin-deficient *ob/ob* mice during desynchronized feeding. (**A**) Body weight gain over the six-week period. Desynchronized Feeding (DF) and Synchronized Feeding (SF) begins on Day 1 as noted by the y-axis. No significant differences were observed in weight gain within leptin-deficient *ob/ob* mice. (**B**) Total activity over the six-week period as measured by IR-beam breaks. No significant differences were observed in total cumulative activity between DF and SF groups. However, the DF group had significantly less activity during the circadian dark phase as compared to SF group (*t*(14) = −2.50, p = 0.03).

**Figure 2:**
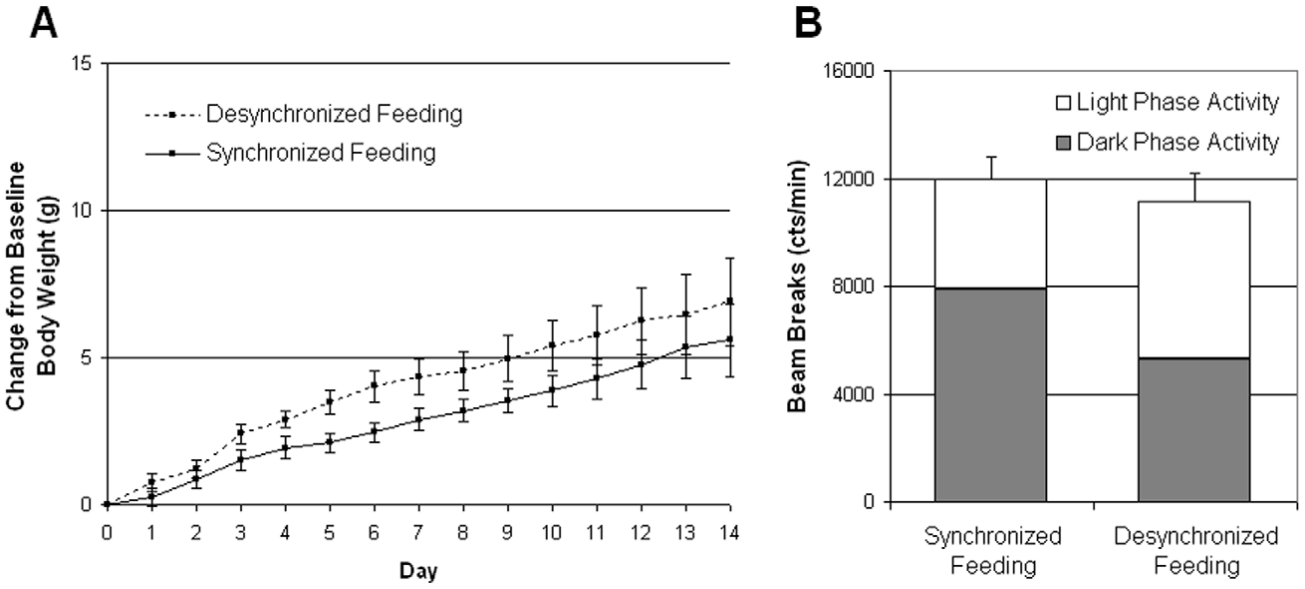
Experiment 2: Body weight gain and total activity in *ob/ob* mice treated with constant leptin during desynchronized feeding. (**A**) Body weight gain over the two-week period. No significant differences were observed in weight gain in *ob/ob* mice treated with constant non-rhythmic leptin. (**B**) Total activity over the two-week period as measured by IR-beam breaks. No significant differences were observed in total cumulative activity between DF and SF groups. While not statistically significant, the SF group appears to be more active during the circadian dark phase as compared to DF group.

**Figure 3:**
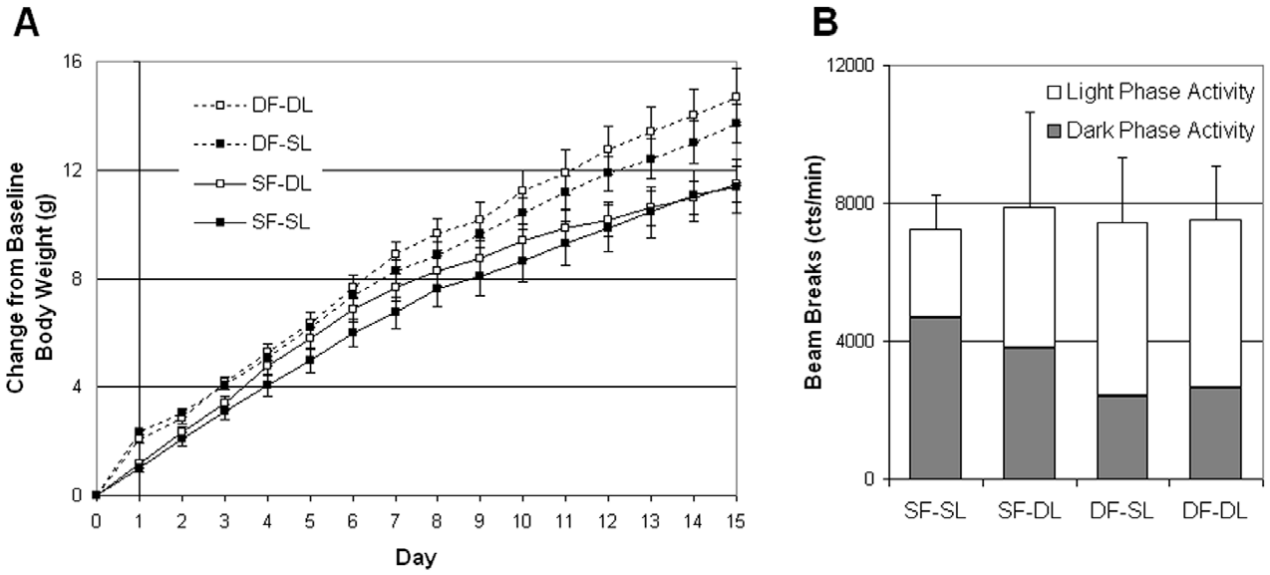
Experiment 3: Body weight gain and total activity in *ob/ob* mice given a single leptin injection during desynchronized feeding. (**A**) Body weight gain over the two-week period. *Ob/ob* mice given rhythmic leptin, in the form of a once daily injection, gain more weight during DF than SF (F(1, 30) = 9.09, p = 0.01). Furthermore, of the four groups (F(3, 28) = 3.21, p = 0.04) *ob/ob* mice within the most circadian desynchronized DF-DL group gained the most weight, whereas the most synchronized SF-SL group gained the least amount of weight. (**B**) Total activity over the two-week period as measured by IR-beam breaks. No significant differences were observed in total cumulative activity among the groups. However, a trend was noted for an increase in circadian dark phase activity (F(1, 30) = 4.01, p = 0.05) within the SF groups compared to the DF groups. Abbreviations: Desynchronized Feeding with Desynchronized Leptin (DF-DL), Desynchronized Feeding with Synchronized Leptin (DF-SL), Synchronized Feeding with Desynchronized Leptin (SF-DL), Synchronized Feeding with Synchronized Leptin (SF-SL). Symbols: DF  =  dotted lines, SF  =  solid lines, DL  =  open squares, SL  =  filled squares.

Here we present results indicating that a circadian rhythm of leptin is necessary for increased weight gain from desynchronized feeding (DF) in mice (e.g. light-phase feeding). As our previous study demonstrated [8], wild type B6 mice gain excessive weight when fed a high-fat diet during the light-phase (DF) compared to groups fed the same diet only during the circadian dark phase (synchronized feeding, SF). Contrary to wild type B6 mice, leptin deficient *ob/ob* mice do not gain excess weight during DF (experiment 1, Figure 1a). Since the primary difference between the *ob/ob* and the B6 mouse is the presence of leptin, we next gave *ob/ob* mice leptin using a continuous release leptin pump (experiment 2). Interestingly, continuous leptin failed to cause excessive weight gain during DF (Figure 2a). However, when treated with once daily leptin injections, to mimic a rhythmic leptin profile, *ob/ob* mice respond like wild type B6 mice and gain excessive weight during DF (experiment 3, Figure 3a).

### Experiment 1. Leptin deficient *ob/ob* mice fail to gain excess weight during desynchronized feeding

To test the hypothesis that leptin may be central to the weight gain seen during ‘wrongly’ timed feeding, leptin-deficient *ob/ob* mice were placed on DF and monitored for body weight, food intake, and activity levels. To control for the length of feeding and fasting periods, DF *ob/ob* mice are compared to synchronized feeding (SF) *ob/ob* mice that have access to food for the same length of time but only during the normal circadian feeding period (e.g. the dark phase).

#### Body Weight

Body weights of untreated leptin-deficient *ob/ob* were similar under both DF and SF conditions. The timing of the high-fat diet failed to affect body weight gains in leptin deficient *ob/ob* mice (Figure 1a, F(1, 14) = 1.07, p = 0.32). Importantly, this result is contrary to what is observed in the wild type B6 where we demonstrate that caloric intake occurring during the ‘wrong’ time of day leads to increased weight gain [8]. These data suggest that the presence of leptin may play a key role in weight gain from ‘wrongly’ timed feeding since the absence of leptin leads to equal weight gains in *ob/ob* mice under both DF and SF conditions.

As expected, both DF and SF *ob/ob* mice have a significant increase in body weight over the course of the study (F(14, 196) = 1306.1, p<0.001) indicating that all *ob/ob* mice gain weight while on the high-fat diet.

#### Caloric Intake

No differences were seen in overall weekly caloric intake between DF and SF *ob/ob* mice (F(1, 13) = 0.49, p = 0.50). Similarly, no differences were seen in cumulative caloric intake between DF (980±13 kcal) and SF (1002±30 kcal) *ob/ob* mice over the six week period (*t*-Test, *t*(8) = −0.67, p = 0.52). (Table 1)

**Table 1:** Caloric intake data from experiment 1.

		Week 1	Week 2	Week 3	Week 4	Week 5	Week 6	2-week Cumulative	6-week Cumulative
DF	**Average**	**201**	**179**	**162**	**156**	**148**	**133**	**380**	**980**
	StDev	13	13	8	13	7	8	23	37
	*StErr*	*5*	*5*	*3*	*5*	*3*	*3*	*8*	*13*
SF	**Average**	**211**	**169**	**183**	**163**	**150**	**128**	**379**	**1002**
	StDev	16	28	14	22	15	8	42	80
	*StErr*	*6*	*10*	*5*	*8*	*5*	*3*	*15*	*30*

Standard deviation (StDev) and standard error (*StErr*) of Desynchronized Feeding (DF) and Synchronized Feeding (SF).

#### Activity

Over the course of the six week experiment, no differences were seen in cumulative activity counts (Figure 1b, *t*-Test, *t*(14) = 0.51, p = 0.62). However, there was significant difference in the amount of activity occurring during the circadian dark phase: the DF group had significantly less activity during the dark phase than the SF group (*t*-Test, *t*(14) = −2.50, p = 0.03). This decrease in dark activity was not accompanied by a statistically significant increase in light phase activity within the DF group most likely due to the large within group variation (*t*-Test, *t*(14) = 0.85, p = 0.41).

### Experiment 2. Leptin deficient *ob/ob* mice fail to gain excess weight during desynchronized feeding when provided with non-rhythmic, continuous leptin treatment

To test the hypothesis that the presence of leptin alone could lead to excessive weight gains from DF in the *ob/ob* mouse, leptin was provided non-rhythmically by a continuous release osmotic pump (described in methods) for two weeks. This length of time was chosen based on previous data indicating that B6 mice on DF gain significantly more weight than SF mice within two weeks [8].

#### Body Weight

When treated with non-rhythmic continuous leptin, DF *ob/ob* mice weighed the same as SF *ob/ob* mice suggesting that the presence of leptin alone was not sufficient to lead to weight gain from DF as seen in wild type mice. The timing of feeding did not affect body weight in *ob/ob* mice provided non-rhythmic leptin (Figure 2a, F(1, 12) = 2.314, p = 0.15). As expected, a significant increase in body weight was seen in both groups over the course of the study (F(13, 156) = 27.23, p<0.001) indicating that *ob/ob* mice with non-rhythmic leptin still gain weight while on the high-fat diet.

#### Caloric Intake

No differences were seen in overall weekly caloric intake between DF and SF *ob/ob* mice (F(1, 12) = 0.42, p = 0.53). Similarly, no differences were seen in the cumulative caloric intake between DF (251±11 kcal) and SF (263±14 kcal) *ob/ob* non-rhythmic leptin treated mice over the two week period (*t*-Test, *t*(12) = −0.65, p = 0.53). (Table 2)

**Table 2:** Caloric intake data from experiment 2.

		Week 1	Week 2	2-week Cumulative
DF	**Average**	**134**	**118**	**251**
	StDev	12	22	30
	*StErr*	*5*	*8*	*11*
SF	**Average**	**144**	**119**	**263**
	StDev	13	30	38
	*StErr*	*5*	*11*	*14*

Standard deviation (StDev) and standard error (*StErr*) of Desynchronized Feeding (DF) and Synchronized Feeding (SF).

#### Activity

Over the course of the two week experiment, no differences were seen in cumulative activity counts (Figure 2b, *t*-Test, *t*(12) = -0.64, p = 0.53). Similarly, no statistical differences were observed in the amount of activity occurring during the circadian light phase (*t*-Test, *t*(12) = 1.23, p = 0.24) or the circadian dark phase (*t*-Test, *t*(12) = −1.40, p = 0.19). However, as in the previous experiment, the SF group appears to favor being active during the circadian dark phase more than the DF group.

These data indicate that the presence of leptin alone is not sufficient to cause excessive weight gain from DF.

### Experiment 3. Excessive weight gain during desynchronized feeding occurs when leptin deficient *ob/ob* mice are given rhythmic leptin treatment

To test the hypothesis that rhythmic leptin is necessary to lead to excessive weight gain from DF, we gave a once daily leptin injection to *ob/ob* mice (see methods). In addition to examining the effects of feeding time (e.g. the DF and SF groups), we also examined the timing of the leptin injection. Half of each feeding group were given a leptin injection during the feeding phase to produce a naturally occurring feeding-associated leptin peak (synchronized leptin  =  SL) while the other half of the feeding group were given a leptin injection during the fasting period to produce an abnormally occurring leptin peak desynchronized from feeding (desynchronized leptin  =  DL). Therefore, the four experimental groups (each N  =  8) were as follows:

1. SF-SL, having normally timed feeding and a normal feeding-associated leptin peak,
2. SF-DL, having normally timed feeding and a feeding-dissociated leptin peak,
3. DF-SL, having ‘wrongly’ timed feeding and a normal feeding-associated leptin peak, and
4. DF-DL, having ‘wrongly’ timed feeding and a feeding-dissociated leptin peak.

#### Body Weight

Only when leptin was given by daily injection did DF *ob/ob* mice gain significantly more weight than SF *ob/ob* mice (Figure 3a, F(1, 30) = 9.09, p = 0.01). This suggests that a rhythm of leptin is needed in order to gain excessive weight from ‘wrongly’ timed feeding. Further analysis of the four groups (F(3, 28) = 3.21, p = 0.04) demonstrates that *ob/ob* mice within the most circadian desynchronized DF-DL group gained the most weight and were significantly different from the most synchronized SF-SL group (p = 0.01) and the moderately synchronized SF-DL group (p = 0.04). Additionally, DF-SL mice gained more than SF-SL mice (p<0.05). When focusing on the weight gains at the end of the two week study (one-way ANOVA, F(3, 28) = 3.55, p = 0.03), DF-DL gained significantly more than SF-SL (p = 0.01) and SF-DL mice (p = 0.01). Additionally, DF-SL mice showed a trend to gain more than SF-DL (p  =  0.08) and SF-SL mice (p = 0.07), suggesting that a desynchronized feeding rhythm leads to more metabolic impairments than a desynchronized leptin rhythm.

Interestingly, leptin injection time by feeding-association (DL vs. SL) alone had no effect on body weight gains in *ob/ob* mice (F(1, 30) = 0.71, p = 0.41) suggesting that a desynchronized leptin rhythm by itself would not lead to significant weight change without the additional component of ‘wrongly’ timed feeding. However, the timing of the leptin peak may influence body weight to some degree as represented by the beginning of a separation of body weight gains between the DF-DL and DF-SL groups.

As expected, all groups gained weight over the duration of the study (F(14, 392) = 601.42, p<0.001). There was a significant interaction effect (F(14, 392) = 5.4722, p<0.001) between feeding phase and study duration suggesting that the DF *ob/ob* mice gain weight differently over the two week period than SF *ob/ob* mice regardless of leptin injection time.

#### Caloric Intake

Of the four groups, no differences were seen in overall weekly caloric intake (F(3, 28) = 1.50, p = 0.24). Similarly, an one-way ANOVA of the cumulative caloric intake over the two week period failed to show any differences in caloric intake (F(3, 28) = 1.50, p = 0.24) among the four groups (SF-SL 203±9 kcal; SF-DL 214±10 kcal; DF-SL 206±10 kcal; DF-DL 188±4 kcal). Neither feeding time (F(1, 28) = 1.78, p = 0.19), leptin injection time (F(1, 28) = 0.13, p = 0.72), or their interaction (F(1, 28) = 2.60, p = 0.12) had an effect on caloric intake. (Table 3)

**Table 3:** Caloric intake data from experiment 3.

		Day 1–2	Day 3–4	Day 5–6	Day 7–8	Day 9–10	Day 11–12	Day 13–14	Week 1	Week 2	2-week Cumulative
SF-SL	**Average**	**31**	**31**	**30**	**28**	**27**	**25**	**30**	**106**	**97**	**203**
	StDev	2	4	5	5	3	4	7	12	16	26
	*StErr*	*1*	*1*	*2*	*2*	*1*	*1*	*3*	*4*	*6*	*9*
SF-DL	**Average**	**35**	**34**	**35**	**29**	**28**	**26**	**27**	**119**	**95**	**214**
	StDev	4	4	5	6	6	6	3	14	18	29
	*StErr*	*1*	*1*	*2*	*2*	*2*	*2*	*1*	*5*	*6*	*10*
DF-DL	**Average**	**25**	**29**	**30**	**28**	**27**	**24**	**25**	**98**	**90**	**188**
	StDev	2	2	3	2	2	3	4	6	9	11
	*StErr*	*1*	*1*	*1*	*1*	*1*	*1*	*1*	*2*	*3*	*4*
DF-SL	**Average**	**27**	**32**	**33**	**28**	**30**	**27**	**28**	**106**	**99**	**205**
	StDev	2	5	6	6	5	5	4	14	15	28
	*StErr*	*1*	*2*	*2*	*2*	*2*	*2*	*1*	*5*	*5*	*10*

Standard deviation (StDev) and standard error (StErr) of Desynchronized Feeding with Desynchronized Leptin (DF-DL), Desynchronized Feeding with Synchronized Leptin (DF-SL), Synchronized Feeding with Desynchronized Leptin (SF-DL), Synchronized Feeding with Synchronized Leptin (SF-SL).

#### Activity

Over the course of the experiment, no differences were seen in cumulative activity counts (Figure 3b, F(3, 28) = 0.02, p>0.99). Similarly, no statistical differences we observed in the amount of activity occurring during the circadian light phase (F(3, 28) = 0.50, p = 0.68) or the circadian dark phase (F(3, 28) = 1.45, p = 0.25) among the four groups. However, when comparing DF and SF groups, a trend was noted for an increase in circadian dark phase activity (F(1, 30) = 4.01, p = 0.05) among the SF groups.

Together, these data indicate that a leptin rhythm is necessary for the excessive weight gain from DF.

## Discussion

‘Wrongly’ timed feeding, as from DF, has been previously described to lead to excessive weight gains in rodents [7], [8], [9], [22]. The mechanisms leading to this feeding-time dependent weight gain are unknown but we hypothesize that the endogenous leptin rhythm itself may influence weight gain due to its interconnections with body weight regulation [10], [11], [12], feeding behavior [13], [14] and circadian rhythms [15], [16], [19].

**Figure 4:**
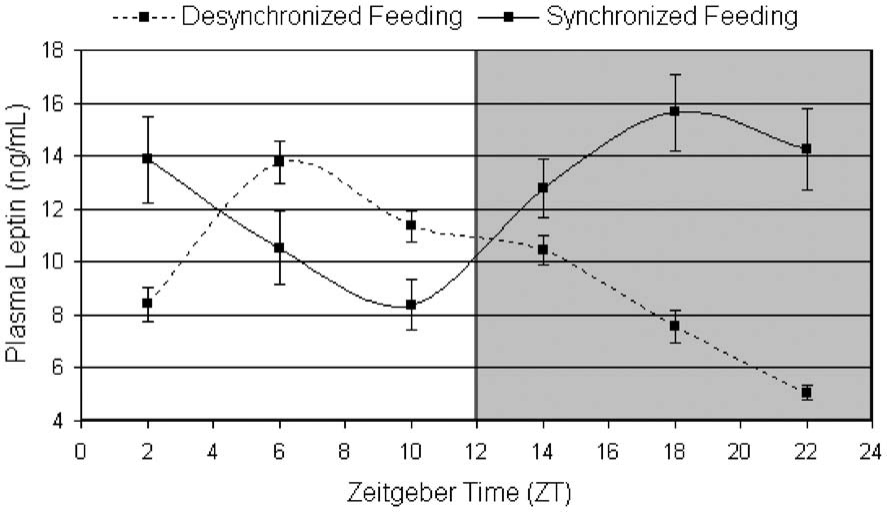
Circulating plasma leptin levels in wild type C57Bl/6J mice during desynchronized (DF) and synchronized feeding (SF). Wild type mice on DF (dotted line) have a leptin peak mid-feeding at ZT 6 followed by an increased drop in leptin during the fasting phase compared to SF mice (solid line) that display a leptin peak at ZT 18. Shaded background represents the circadian dark phase, open background represents the circadian light phase. Abbreviation: Zeitgeber Time (ZT).

In addition to the feeding rhythm (DF vs. SF) in the present study, we also integrated differences in feeding time with the rhythmic or non-rhythmic behavior of the metabolic hormone leptin. Notably, we examine two circadian aspects of leptin expression: 1) rhythmic vs. non-rhythmic expression and 2) synchrony vs. desynchrony with feeding behavior. Within normal, healthy humans and rodents, leptin is naturally rhythmic and associated with feeding time (equivalent to the SF-SL group in experiment 3). In obese individuals, leptin levels are elevated but the rhythm of leptin remains relatively unchanged with the exception of a reduction in amplitude [23], [24]. Wild type mice on DF (equivalent to the DF-SL injection group in experiment 3) however have a significant change in peak phase (12 hours) and a lower leptin trough compared to SF mice (Figure 4). This scenario may be similar to a human shift-worker who must be active and eating during the ‘wrong’ circadian phase. Periods of desynchronized leptin (DL) are much less common in the mouse and human population, but may occur during small windows of entrainment time, such as when adjusting to a new feeding schedule or when experiencing jet-lag.

**Figure 5:**
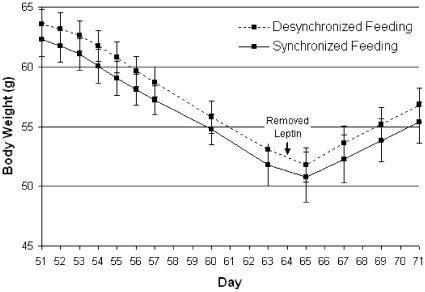
Body weights of *ob/ob* mice treated with constant leptin six weeks into desynchronized (DF) or synchronized feeding (SF). The addition of constant non-rhythmic leptin (at Day 51) after six weeks of feeding does not lead to differential weight loss between the DF (dotted line) and SF (solid line) groups. Removal of leptin (Day 64) after two weeks of treatment does not lead to differential body weight gains between the DF and SF groups.

In experiment 1 and 2, we focus on the rhythmic vs. non-rhythmic expression pattern of leptin. Our data indicate that DF does not lead to excessive weight gains in animals with non-rhythmic leptin profiles, including untreated, leptin-deficient *ob/ob* mice (Figure 1a) and continuously leptin-treated *ob/ob* mice (Figure 2a). In a follow-up study, giving continuous leptin after six weeks of DF, replicated these results (Figure 5), indicating that continuous leptin treatment also does not lead to differential weight loss during ‘wrongly’ timed feeding.

Treating *ob/ob* mice with continuous leptin essentially returns the *ob/ob* mouse to the wild type state with one major exception – continuous leptin treatment does not mimic the natural circadian peaks and troughs within the leptin expression profile. Indeed, B6 mice on DF have leptin peaks associated with feeding behavior as opposed to the light:dark cycle (Figure 4). This association with meal timing has been previously shown in both rodent and human studies [13], [14]. These data from experiments 1 and 2 suggest that the presence of leptin alone is not sufficient to lead to excessive weight gains from ‘wrongly’ timed feeding.

Given that non-rhythmic leptin failed to increase weight gain during DF, we next examined if the presence of a leptin rhythm could lead to increased weight gains during DF. To create a leptin rhythm in the *ob/ob* mice, DF was used in combination with once daily leptin injections (experiment 3). When rhythmic leptin was present, *ob/ob* mice gained excessive weight during DF. As the endogenous leptin profile of wild type mice is also rhythmic, it is possible that the circadian expression of leptin provides additional cues leading to excessive body weights from DF. The data from experiment 3 indicate that a rhythmic profile of leptin is needed to induce weight gains from DF. As an alternative explanation, these data also suggest that rhythmic leptin administration is less effective at preventing weight gain than continuous leptin administration. Indeed, *ob/ob* provided continuous leptin gain less weight over time than *ob/ob* mice given the same amount of leptin in a single daily dose.

**Figure 6:**
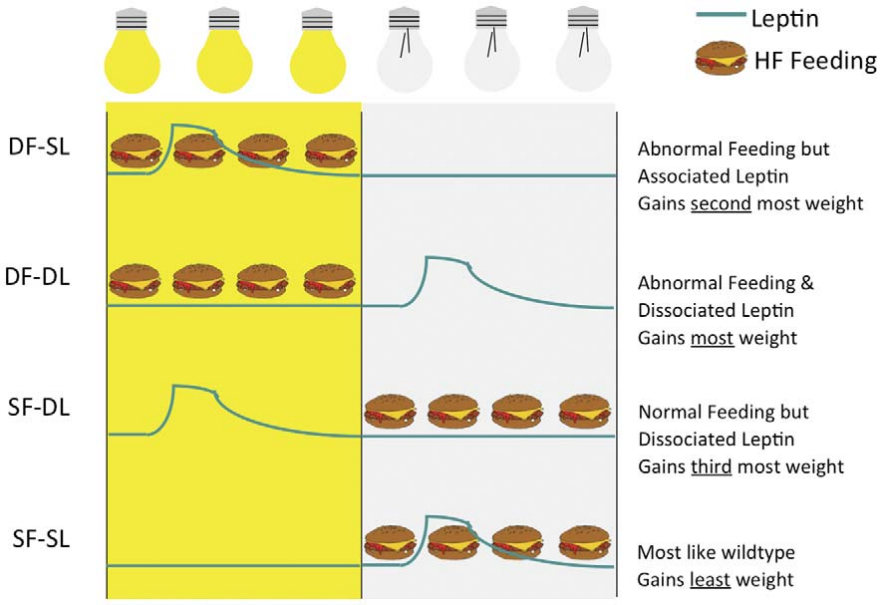
Summary cartoon depicting the results of experiment 3. *Ob/ob* mice fed in the light phase and receiving leptin in the dark phase (DF-DL) gain the most amount of weight over the 2-week period. Conversely, *ob/ob* mice fed in the dark and receiving leptin in the dark (SF-SL) gain the least amount of weight. Of the intermediate groups, (SF-DL and DF-SL), *ob/ob* mice feeding during the light phase gain more weight. Cheeseburgers represent the availability of the high fat diet. Blue lines represent plasma leptin levels within the groups.

Since leptin naturally peaks in association with feeding time, we additionally examined synchronous vs. desynchronous leptin rhythms in relation to feeding time, to determine if this level of desynchrony could also affect body weights during DF. We hypothesized that if ‘wrongly’ timed feeding could lead to weight gain, perhaps ‘wrongly’ timed leptin peaks would lead to exaggerated weight gain. *Ob/ob* mice fed in the dark and receiving a leptin injection during the dark (SF-SL) would closely mirror a normal wild type mouse which has its feeding behavior synchronized to the light:dark cycle and its leptin rhythm synchronized to the feeding behavior. At the opposite extreme is the DF-DL group that was fed exclusively during the ‘wrong’ phase (e.g. light phase) and received leptin injections dissociated from the feeding time (e.g. during the dark phase). Intriguingly, these two groups showed the most profound differences in body weight with the DF-DL mice gaining the most weight and the SF-SL mice gaining the least amount of weight over time. These data suggest that minimal circadian disorganization can lead to optimal body weight regulation whereas maximal circadian disorganization can lead to the greatest impairments in body weight regulation (summarized in Figure 6).

While leptin in known to acutely inhibit food intake in rodents, the data from experiment 3 indicate that leptin’s inhibitory effects are not leading to the observed body weight differences between the groups. Indeed, if the acute effects of leptin were primarily affecting body weight more so than circadian timing effects, one would expect similar body weights between the groups receiving leptin during their feeding phase (e.g. SF-SL and DF-SL groups). However, we observe increased body weight (p<0.05) in mice eating during the light phase (and receiving leptin at that time) than in mice eating during the dark phase (and receiving leptin at that time).

Statistical analyses of the two variables “feeding synchrony” and “leptin synchrony,” indicate that only feeding synchrony will lead to differential weight gain during DF. This suggests that circadian mistimed feeding will lead to a greater impairment of body weight regulation than a mistimed leptin peak. This in conjunction with experiments 1 and 2 suggests that while a rhythm of leptin is necessary to induce increased weight gains from ‘wrongly’ timed feeding, the actual time of the leptin peak is not essential. This is intriguing and mirrors other rhythmic hormones such as GnRH, which is known to have little signaling effect when given continuously but signals robustly when given rhythmically, regardless of the circadian time of day that rhythm is given [25].

It is interesting to note that the mice receiving the once daily leptin injection consumed less calories but gained more weight than the mice receiving the constant release leptin pump. This relative decrease in caloric intake may be due to the presence of the injection (of either leptin or saline) during the feeding phase which leads to increased arousal, exploratory activity, and other stereotypical behaviors including an acute affect on feeding. The relative increase in body weight within the leptin injection mice suggests a different utilization of calories within the leptin injection mice, one that intensely favors weight gain and possibly fat storage.

The rising and falling of leptin levels over the 24-hour day trigger fluctuations in other feeding hormones such as ghrelin [26] as well as fluctuations in leptin receptor (e.g. the long-form of the leptin receptor, Ob-Rb) expression which communicate circulating leptin levels to the brain and in turn influence feeding, circadian rhythms, and also feedback on leptin itself. During a short fast, leptin levels decrease [27] and leptin receptor number and expression are up-regulated helping to promote feeding [28], [29]. With increasing leptin levels, as occurs during re-feeding, leptin receptor number and expression are then down-regulated [30], [31]. We hypothesize that by using a subcutaneous low-dose constant release leptin pump, leptin receptors fail to receive the necessary fluctuations required for leptin receptor number and expression regulation [30] and therefore remain in a down-regulated state which favors body weight maintenance and negative energy balance. We hypothesize that the fluctuations in leptin receptor number and expression are necessary for the increased weight gains observed in mice feeding during the ‘wrong’ circadian phase. Indeed, *ob/ob* mice having no leptin or receiving non-rhythmic leptin do not gain increased body weight from ‘wrongly’ timed feeding. Within a wild type mouse, for example, fluctuations in leptin and leptin receptor number and expression naturally occur coincident with feeding, however when leptin fluctuations occur during ‘wrongly’ timed feeding increased weight gain follows, perhaps through interactions with food-driven mechanisms and/or other circadian-driven mechanisms. Indeed, other research has hypothesized that a major role the leptin circadian rhythm may be to maintain a set threshold of leptin to order to prevent excessive caloric intake [32].

Our results also indicate that despite both DF and SF mice receiving the same length of fasting (12 hours), fasting during the dark phase (and feeding during the light) leads to a greater drop in leptin than fasting during the light (and feeding during the dark; Figure 4). This suggests that the circadian timing of feeding/fasting has an effect on leptin rhythms and moreover that mistimed feeding rhythms, in relation to the light:dark cycle, will result in an increased drop in fasting leptin levels which could lead to an increased up-regulation in leptin receptors, thus favoring positive energy balance and weight gain. In summary, feeding during the wrong circadian phase leads to a greater drop in leptin during the fasting period (Figure 4), which in turn may lead to greater fluctuation and up-regulation of leptin receptors and promote a positive energy balance. The circadian expression of leptin appears important for the cycling of hormonal, behavioral rhythms, and health and this may be due in part to daily fluctuations in leptin receptor number and expression.

It is important to highlight that the *ob/ob* mouse used in this study is, by itself, a metabolic model that develops without leptin and becomes grossly obese. However, given the importance of carefully and accurately controlling the leptin rhythm, we chose to use the *ob/ob* mouse in order to experimentally control the leptin rhythm completely. To alleviate some of the expected obesity in this model, *ob/ob* mice began the experiment at 6-weeks of age when their body weights were relatively reduced (∼30g). However, it is possible that confounding metabolic factors present in the *ob/ob* mouse may lead to misinterpretation of the data. Therefore, additional experiments using wild type mice or another non-metabolic model would make ideal follow-up studies.

To our knowledge, these data present the first evidence that leptin has differential effects depending on a peak in leptin levels. One previous study did examine the treatment effects of a leptin injection given in the morning, evening, or both in the morning and evening in obese humans [20]. However, no differences were seen in weight loss among the groups. Importantly, this study used obese individuals, a population which is now mostly considered to be leptin ‘resistant’. To our knowledge, no study has examined the role of the circadian leptin rhythm in non-obese individuals’ health and our study is the first to present data which suggests that leptin’s once daily peak in expression may be an important signal for body weight regulation particularly involved in ‘wrongly’ timed feeding.

It is interesting to integrate our results from an evolutionary prospective. It would be maladaptive, for instance, for a nocturnal animal to be awake and seeking food during the daytime when predators are readily available. But if a nocturnal animal critically requires calories, it may override the circadian clock (therefore being awake and eating during the ‘wrong’ circadian time) and it would then be adaptive to utilize the forged calories in such a way so to limit future food seeking behavior during the ‘wrong’ time. This can be accomplished by storing the calories and increasing body weight. An increase in body weight is precisely what happens during desynchronized feeding and it could be the endogenous leptin rhythm coupled to the feeding time that promotes such a response. Perhaps once a beneficial response promoting fitness, body weight gain from ‘wrongly’ timed feeding now has detrimental implications for many humans consuming a significant portion of their calories during non-ideal circadian times, including non-breakfast eaters [33] and night-shift workers [5] both of which an increased risk for weight gain and obesity.

## Materials and Methods

All experimental mice were housed and handled according to the Federal Animal Welfare guidelines. All studies were conducted at Northwestern University using procedures approved in advance by the Institutional Animal Care and Use Committee, university assurance number A3283-01.

### Animals

Male *ob/ob* mice were ordered from Jackson Laboratory (Bar Harbor, ME) at 4–5 weeks of age and immediately singly-housed within temperature, light, and humidity controlled light boxes on a 12 hour light, 12 hour dark schedule. Mice begin experimentation at 6-weeks of age after having one week of environmental acclimation. On average, *ob/ob* mice were ∼30g when beginning the experiment. Male C57BL/6J (B6) mice were bred at Northwestern University and began experimentation at 10–11 weeks old within temperature, light, and humidity controlled light boxes on a 12 hour light, 12 hour dark schedule. On average, B6 mice were ∼24.5 g when beginning the experiment.

### Diets

All mice were initially fed a standard chow (LabDiet #5K52, JL Rat and Mouse/Auto 6F, Arlington Heights, IL distributor) of which 16% of the calories are from fat until the start of the desynchronized feeding protocol. After the start of desynchronized feeding, mice were fed a high-fat diet in which 60% of the calories are from fat (Research Diets #D12492, New Brunswick, NJ). A subset of *ob/ob* mice were allowed continuous free-access to food and were maintained on the standard chow diet in order to limit weight gain during ad libitum feeding.

*Desynchronized Feeding*. Mice were placed onto a restricted feeding protocol as previously described [8]. Desynchronized Feeding (DF) mice had unlimited access to the high-fat diet during the 12-hour circadian light phase only, during the 12-hour dark phase no food was provided. Synchronized Feeding (SF) mice were given the opposite feeding schedule, receiving unlimited high-fat diet during the 12-hour dark phase but no food was provided during the 12-hour light phase. At all times, all mice had free access to water. Feeding conditions were maintained by manually switching each mouse every 12 hours between the individual’s habituated light cage and the individual’s habituated dark cage.

### Leptin pumps

14-day constant release leptin pumps (Alzet mini-osmotic pump #1002, Cupertino, CA) were filled with a 100ug/kg/day leptin solution containing 0.9% saline solution. Pumps were activated in a warm saline bath for 12 hours prior to implantation. Pumps were implanted subcutaneously into isofluane-anesthetized mice in the dorsal interscapular region just prior to their designated feeding phase. Previous studies have indicated that an osmotic pump delivers leptin stably and consistently [34]. Leptin pumps were surgically removed 14 days after implantation.

### Leptin injections

**Figure 7:**
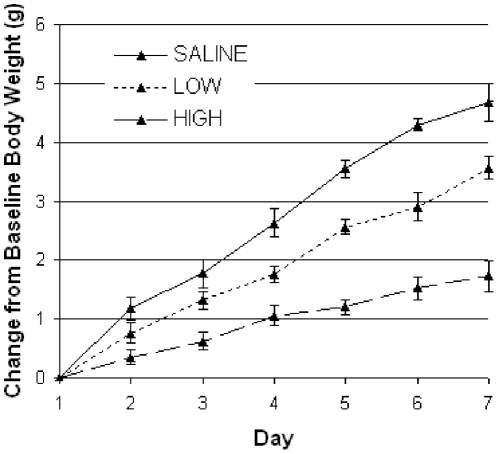
Body weight gains of *ob/ob* mice administered two different doses of leptin or saline over 7 days. The low-dose of leptin injection (100 ug/kg/day) was sufficient to reduce body weight in *ob/ob* mice (dotted line) compared to the saline control (solid line). The high-dose of leptin injection (1 mg/kg/day) greatly reduced weight gain (dashed) compared to saline control.

**Figure 8 pone-0025079-g008:**
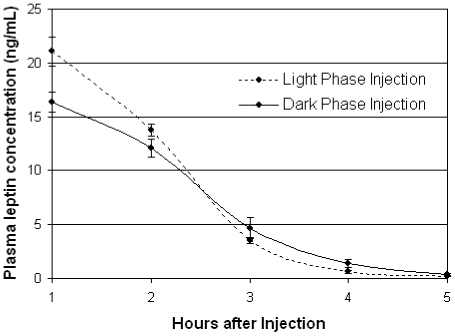
Circulating plasma leptin levels hours after low-dose leptin injection. After the 11^th^ 100 ug/kg/day injection, circulating leptin was found to be absent from the plasma of *ad libitum* fed animals within 4–6 hours regardless of whether the injection occurred during the circadian light phase at ZT 5 (dotted line) or during the circadian dark phase at ZT 17 (solid line).

**Figure 9:**
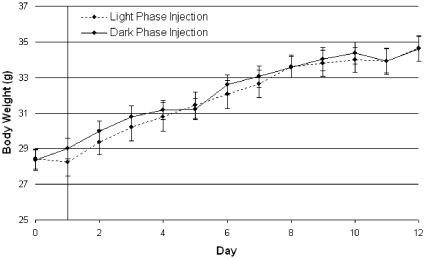
Body weights of *ob/ob* mice fed *ad libitum* and treated with a once daily low-dose leptin injection. When *ob/ob* mice are allowed free access to food, a single 100 ug/kg/day leptin injection during the circadian light at ZT 5 (dotted line) or during the circadian dark phase at ZT 17 (solid line) does not result in differential weight gain.

Subcutaneous leptin injections to the dorsal interscapular region were given once daily at either ZT4 (i.e. four hours after light onset) or ZT16 (i.e. four hours after dark onset) at the same dose as the leptin pumps, 100 ug/kg/day. Both the dose and the timing of the leptin injections were chosen to mirror the physiological leptin levels of wild type mice fed the same SF or DF protocol. The single 100 ug/kg/day leptin dose was determined sufficient to slow weight gain while on a high-fat diet (Figure 7), to be absent from circulating plasma 4–6 hours post injection (Figure 8), and to elevate leptin to wild type physiological levels (Figure 4). Since DF wild type mice experience the highest physiological levels of leptin from ZT4-ZT10 and SF wild type mice experience the highest physiological levels of leptin from ZT16-ZT22 (Figure 4), ZT4 and ZT16 were chosen as the injection times to elevate leptin in *ob/ob* mice to wild type levels during the same 4-6 hr period similarly fed wild type would experience a physiological leptin peak. A saline injection was given at the counter ZT hour to control for the stress of injection at both ZT4 and ZT16. It was previously determined that the timing of the leptin injection alone while on *ad libitum* standard chow feeding did not lead to differences in body weight (Figure 9).

*Activity*. Overall activity was recorded continuously using a one-dimensional, three-beam infrared (IR) monitoring board. Beam breaks were recorded and data analyzed using an online recording program (*Clocklab*, Actimetrics, Wilmette, IL).

### Statistical Analysis

Unless otherwise stated, data were analyzed using a repeated measured ANOVA with the aid of a statistical program (StatsSoft Statistica, Tulsa, OK).

### Experiment 1

*Ob/ob* mice were randomly separated into two groups: DF (N  =  8) and SF (N  =  8). Each group continued on the designated feeding schedule for six weeks during which time body weights were taken regularly at ZT0 and ZT12 corresponding to food onset and offset. Caloric intake during the light and dark period was measured weekly by manual food removal.

### Experiment 2

*Ob/ob* mice were randomly separated into two groups: DF (N  =  7) and SF (N  =  7). Before the onset of restricted feeding, mice were implanted with a leptin pump 24 hours prior to the first high-fat feeding period. Each group continued on the designated feeding schedule for two weeks during which time body weights were taken regularly at ZT0 and ZT12 corresponding to food onset and offset. Caloric intake during the light and dark period was measured weekly by manual food removal.

### Experiment 3

*Ob/ob* mice were randomly separated into four groups: SF-SL (N  =  8), SF-DL (N  =  8), DF-SL (N  =  8) and DF-DL (N  =  8). Mice begin leptin injections 24 hours prior to the onset of restricted feeding. All groups continued on the designated feeding schedule for two weeks during which time body weights were taken regularly at ZT0 and ZT12 corresponding to food onset and offset. Caloric intake during the light and dark period was measured every 48 hours by manual food removal.

### Supplemental Experiments (Figures 4,5,7–9)

To determine endogenous leptin levels (Figure 4), wild type mice were randomly separated into two groups: DF (N  =  6) and SF (N  =  6). After two weeks of the designated feeding schedule, plasma blood leptin was measured at six points equally spaced over the 24-hour day (ZT 2, ZT 6, ZT 10, ZT 14, ZT 18, and ZT 22).

To determine if constant, non-rhythmic leptin leads to differential weight loss during DF (Figure 5), *ob/ob* mice from Experiment 1 were then implanted with a leptin pump after six weeks of DF and then maintained on the DF feeding schedule for an additional two weeks. Body weights were measured daily for the first week and then every 2–3 days. The leptin pump was surgerically removed after two weeks and body weight gain was measured for an additional week after leptin removal.

To determine if the low-dose leptin injection (100 ug/kg/day) was sufficient to slow body weight gain in *ob/ob* mice (Figure 7), *ob/ob* mice were administered the low-dose of leptin injection (100 ug/kg/day; N  =  5), a high-dose of leptin injection (1 mg/kg/day; N  =  4), or saline (N  =  4) over the course of one week. Body weights were measured once a day just prior to the leptin injection at ZT 11.

To determine the rate of clearance of the low-dose leptin injection in *ob/ob* mice (Figure 8) and if the leptin injection time lead to body weight differences independently of temporal feeding restriction (Figure 9), *ad libitum* fed, ob/ob mice were injected with the low-dose leptin injection at either ZT 5 (N  =  7) or ZT 17 (N  =  6) for two weeks. Body weights were taken daily, 12-hours after the leptin injection. Plasma leptin was measured on the 11^th^ day from 1-5 hours after the leptin injection.
